# Protocolized strategies to encourage early mobilization of critical care patients: challenges and success

**DOI:** 10.62675/2965-2774.20250128

**Published:** 2025-01-30

**Authors:** Patrick Sepúlveda, Adrián Gallardo, Ricardo Arriagada, Eduardo González, Patricia Rieken Macedo Rocco, Denise Battaglini

**Affiliations:** 1 Hospital San Juan de Dios Servicio de Medicina Física y Rehabilitación La Serena Chile Servicio de Medicina Física y Rehabilitación, Hospital San Juan de Dios - La Serena, Chile.; 2 Sanatorio Clínica Modelo de Morón Buenos Aires Argentina Cuidados Respiratorios, Sanatorio Clínica Modelo de Morón - Morón, Buenos Aires, Argentina.; 3 Hospital Las Higueras Unidad de Cuidados Intensivos Talcahuano Chile Unidad de Cuidados Intensivos, Hospital Las Higueras - Talcahuano, Chile.; 4 Hospital San Pablo Unidad de Cuidados Intensivos Coquimbo Chile Unidad de Cuidados Intensivos, Hospital San Pablo - Coquimbo, Chile.; 5 Universidade Federal do Rio de Janeiro Instituto de Biofísica Carlos Chagas Filho Laboratory of Pulmonary Investigation Rio de Janeiro RJ Brazil Laboratory of Pulmonary Investigation, Instituto de Biofísica Carlos Chagas Filho, Universidade Federal do Rio de Janeiro - Rio de Janeiro (RJ), Brazil.; 6 University of Genoa Department of Surgical Sciences and Integrated Diagnostics Genoa Italy Department of Surgical Sciences and Integrated Diagnostics, University of Genoa -Genoa, Italy.

**Keywords:** Critical care, Early ambulation, Quality improvement, Rehabilitation, Respiration, artificial, Physical therapy modalities, Exercise therapy, Length of stay, Patient care planning, Quality of life, Intensive care units

## Abstract

Technological advances and interprofessional teamwork have significantly improved survival rates of critically ill patients. However, this progress has also introduced new challenges, such as intensive care unit-acquired weakness, which can contribute to postintensive care syndrome. Both conditions are associated with increased morbidity and mortality, prolonged length of hospital stay, higher social and health care costs, and reduced quality of life for patients and their families. Timely physical therapy plays a crucial role in mitigating intensive care unit-acquired weakness and postintensive care syndrome. Key recommendations for the effective rehabilitation of patients in the intensive care unit include education and training, communication and collaboration, patient screening, planning of activities, distribution of functions focused on teamwork, patient cooperation, safety assessments, patient positioning, functional mobilization, and documentation of outcomes. This narrative review aims to update the current understanding of the influence of physical therapy and critical care teamwork on intensive care unit patients and to provide evidence-based recommendations for promoting early mobilization in the intensive care unit setting.

## INTRODUCTION

Several complications are associated with bed rest in the intensive care unit (ICU),^(1)^ including muscle weakness, impaired physical functioning, and neurocognitive and psychiatric symptoms; these complications are collectively known as postintensive care syndrome (PICS).^(2)^ ICU patients often develop generalized muscle weakness due to acute illness, which impacts both peripheral and respiratory muscles; this condition is called ICU-acquired weakness (ICUAW). ICUAW affects up to 25% of critically ill patients, and it is associated with high in-hospital morbidity and mortality^(3)^ and can lead to long-term complications beyond the hospital stay.^(4)^ Furthermore, PICS and ICUAW have lasting effects on patients, families, and caregivers.^(5)^

Mobilization, the process of getting patients out of bed and encouraging active movement, is a key element of the "ABCDEF" bundle.^(6)^ When initiated within 72 hours after ICU admission, mobilization combined with appropriate nutritional support can significantly reduce the length of hospital stay.^(7)^ Mobilization and physical therapy protocols, which are enhanced by technological advancements, have been shown to improve survival, decrease morbidity, and shorten the duration of mechanical ventilation (MV) and length of ICU stay.^(8-10)^ Despite its benefits, recent findings from the Treatment of Early Activity and Mobilization (TEAM) trial,^(11)^ which investigated whether increased early mobilization in critically ill patients would lead to better outcomes than standard care, revealed that patients who were randomized to the early mobilization group experienced a higher incidence of adverse events such as desaturation, cardiovascular instability, and accidental removal of medical devices. These findings suggest that not all ICU patients are suitable candidates for early mobilization, highlighting the need for a comprehensive screening process to identify those who are likely to benefit while minimizing risk.

Given these findings, it is crucial to establish evidence-based protocols for safe and appropriate mobilization, particularly for patients receiving MV, renal replacement therapy, or circulatory support.^(6,7)^ This narrative review aims to update the current understanding of mobilization and physical therapy in ICU patients and propose evidence-based strategies for implementation. This review also emphasizes the role of the multidisciplinary ICU team in preventing complications due to prolonged bed rest and highlights the importance of increasing physician awareness. The key steps discussed include education and training, communication and collaboration, patient screening, planning of activities, distribution of functions focused on teamwork, patient cooperation, safety assessments, patient positioning, functional mobilization, and documentation of outcomes.

To conduct a comprehensive and updated narrative review, we analyzed the most recent literature on physical therapy in intensive care by searching the PubMed database for observational studies, randomized trials, systematic reviews, and meta-analyses published up to June 24, 2024. The search terms that were used included the following: ((physical therapy) OR (physiotherapy) OR (rehabilitation) OR (mobilization)) AND ((education) OR (training) OR (collaboration) OR (teamwork) OR (safety) AND (outcome) AND (intensive care)). After removing duplicates and cross-referencing articles, we refined our selection on the basis of novelty, relevance, and English language. Given the narrative nature of this review, our selection was not strictly systematic; instead, we documented the total number of retrieved and screened articles (n = 5,677).

### Education and training

Healthcare professionals in ICU settings should be educated on the importance of early mobilization in daily clinical practice to reduce complications from prolonged MV and shorten length of ICU stay.^(9)^ However, knowledge, attitudes, and practices regarding early mobilization in the ICU remain insufficient. A recent survey revealed that only 2.5% of nurses had a good understanding of the benefits of mobilization and the relevant protocols, 52.3% demonstrated limited knowledge, and 43% did not consider mobilization a priority. Nurses who received appropriate training had better knowledge and attitudes than did those who did not.^(12)^ Another recent study revealed that knowledge among healthcare workers was poor (16.8%), fair (57.9%), and good (25.3%). Physiotherapists with more than 5 years of ICU experience, previous specialized training, and a solid understanding of guidelines were better equipped to prevent PICS and ICUAW by effectively implementing early mobilization.^(13)^

The implementation of protocolized strategies has been effective in increasing activity levels in critically ill patients.^(14,15)^ However, these protocols are only successful if staff fully understand the rationale behind early mobilization. Additional strategies include developing training materials; fostering interdisciplinary collaboration; conducting simulation and practical sessions; offering mentorship programs, workshops, and refresher courses; educating patients and families; implementing feedback mechanisms; and monitoring performance.^(16,17)^

### Communication and collaboration

Effective communication and collaboration among ICU staff are essential for achieving optimal patient outcomes and ensuring patient-centered care. Some key strategies to foster effective communication within the ICU team include the following: 1) regular interdisciplinary meetings and bedside rounds can promote a consistent flow of information; 2) open and transparent communication with patients and families builds trust and allows them to participate in treatment decisions;^(18)^ 3) clear, jargon-free language should be used when communicating with patients and families; 4) effective communication techniques, such as active listening, empathy, and appropriate nonverbal cues, should be applied to ensure accurate message delivery and understanding,^(18)^ and patient-centered care should be prioritized by focusing on patient preferences, values, and goals when creating care plans; and 5) ICU staff should be provided with training on effective communication strategies, cultural sensitivity, and patient-centered care principles.^(16,19,20)^ All healthcare team members should be involved in daily evaluations and discussions about early mobilization. The planning and implementation of such activities should be a collective effort, integrating input from the entire staff.^(21,22)^

### Initial action: patient screening

Contraindications to mobilization must be carefully considered to avoid potential harm or complications. Some common contraindications in the ICU setting include myocardial infarction, active bleeding, increased and unstable intracranial pressure, and unstable pelvic fractures.^(23)^ As reported by the TEAM trial,^(11)^ patients who were randomized to the early mobilization group manifested more adverse events than the usual-care group did, highlighting the need for accurate patient screening before the initiation of mobilization. The key aspects of patient screening derived from the TEAM trial include the following: 1) Clinical Stability: patients must demonstrate hemodynamic stability without significant vasopressor support or signs of active bleeding; 2) Neurological Status: the patient's level of consciousness and ability to follow commands should be assessed. Cooperative patients with minimal sedation are better candidates for safe mobilization. 3) Respiratory Stability: patients should have stable oxygenation and ventilation settings, with consideration for those on noninvasive ventilation (NIV) or low levels of invasive MV. 4) Absence of contraindications: conditions such as uncontrolled intracranial pressure, unstable fractures, and ongoing myocardial infarction are key exclusion criteria. 5) Individualized Risk Assessment: screening should include evaluating the risk of adverse events such as line displacement, falls, or cardiovascular complications during mobilization. The findings of the TEAM trial emphasized that early mobilization should be approached with caution and tailored on the basis of each patient's clinical status.

Suitable candidates for mobilization typically include cooperative adults (aged ≥ 18 years), those on spontaneous ventilation or NIV for ≥ 48 hours, and those without intracranial hypertension or hemodynamic instability.^(24)^ While patients on invasive MV or those with poor cooperation require special attention, they are not absolutely contraindicated for mobilization. Patients with hemodynamic instability or desaturation may need to be excluded from mobilization interventions.^(22)^ The optimal timing, level of activity, and exercise progression (intensity, repetitions, difficulty) remain unclear and require further investigation.^(24,25)^ Therefore, a daily screening process to identify patients who may benefit from mobilization is essential.^(26)^

### Planning of activities

Effective planning for ICU mobilization must consider patient-related, staff-related, resource-related, and structural factors. A safety classification system that evaluates respiratory, neurological, and cardiovascular stability, along with considerations such as renal replacement therapy, should guide decisions about mobilization in or out of bed.^(1)^ Recent clinical practice guidelines emphasize the use of physiological criteria to initiate and discontinue rehabilitation in the ICU.^(25)^

### Distribution of functions focused on teamwork

Effective early mobilization relies on teamwork, with each healthcare professional contributing their specific skills.^(27,28)^ Successful mobilization requires the commitment of all multidisciplinary team members and regular clinical meetings led by team leaders.^(3,29,30)^

### Patient cooperation

Before initiating physical rehabilitation, it is crucial to assess the patient's level of cooperation, which is key for the rehabilitation process. For this evaluation, a panel of experts has recommended the use of the "Standardized Five Questions" (s5Q) scale, which, through five simple questions, allows determination of the subject's level of cooperation.^(23)^ Additionally, the Confusion Assessment Method (CAM)-ICU-7 and the Intensive Care Delirium Checklist (ICDSC) can be used to assess delirium levels, whereas the Richmond Agitation-Sedation Scale (RASS) and pain levels should be evaluated before initiating rehabilitation.^(23)^ Uncontrolled pain and anxiety can cause stress reactions, increase heart rate and blood pressure, and potentially lead to hemodynamic instability.^(31)^ Recognizing that patients may not always be willing or able to engage in physical therapy is essential, as this can be a barrier to performing or continuing physical activity.^(26)^

### Safety assessment

**Figure 1:**
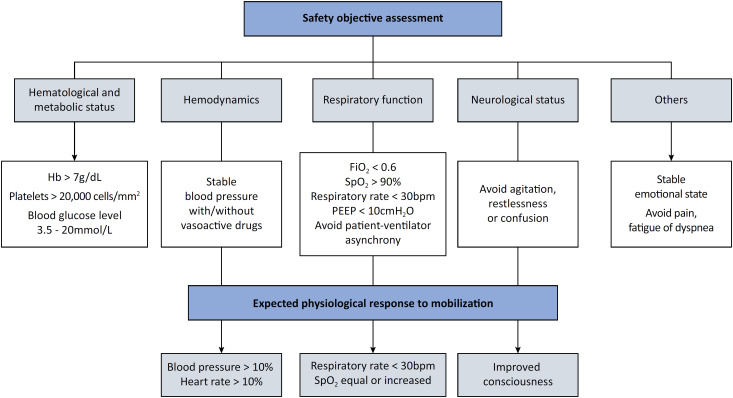
Safety assessment to support mobilization.

Safety is a relevant aspect of any ICU procedure, especially mobilization. Physiological impairments during ICU mobilization can result from excessive physical exertion, increased ventilatory and circulatory demands, fluid and electrolyte imbalances, medication effects, and uncontrolled pain or anxiety.^(30)^ Although the TEAM trial reported more adverse events in the early mobilization group than in the usual-care group,^(11)^ the literature indicates that ICU mobilization is generally feasible and safe, with serious adverse events occurring in fewer than 1% of patients. Premobilization assessments should follow objective criteria^(11)^ (Figure 1).

This flowchart outlines the process for assessing the safety of mobilization in intensive care unit patients. If there are significant changes in hemodynamics (e.g., heart rate changes >25%), respiratory function (e.g., respiratory rate > 35 breaths/minute), or consciousness (e.g., reduced level of consciousness or cooperation), mobilization may need to be contraindicated or halted.

Hb - hemoglobin; FiO_2_ - fraction of inspired oxygen; SpO_2_ - peripheral oxygen saturation; PEEP - positive end-expiratory pressure.

The initial conceptual framework for safety assessment was outlined in 2003.^(31)^ Guidelines suggest that safety assessments should evaluate 1) hemodynamics, 2) respiratory function and airways, 3) neurological status, and 4) other relevant factors.^(1)^ A recent Delphi project reported a consensus on 54 adverse events and 50 risk factors requiring assessment before physical rehabilitation in patients receiving vasoactive drugs.^(32)^

Hemodynamic deterioration during ICU rehabilitation can be caused by excessive activity or inadequate rest. Patients recovering from severe illness or injury may have reduced exercise tolerance or cardiovascular reserve, making them susceptible to tachycardia, hypotension, and decreased organ perfusion. Risks such as orthostatic hypotension, dizziness, nausea, or syncope can occur during early mobilization, with up to 40% incidence reported.^(33)^ Fluid and electrolyte imbalances also contribute to hemodynamic instability by affecting blood volume, pressure, and cardiovascular function.^(34)^ Some medications, such as vasopressors or inotropes, may be needed to maintain blood pressure and cardiac function.^(35)^

Before mobilization, the presence, positioning, and patency of artificial or natural airways (e.g., tracheal tubes or tracheostomies) should be assessed. An artificial airway should not be a contraindication for mobilization if the fraction of inspired oxygen is less than 0.6, provided that there are no other contraindications.^(1)^ A color-coded system (green, yellow, red) based on risk levels is proposed. Mobilization is considered safe if the fraction of inspired oxygen is < 0.6, the peripheral oxygen saturation (SpO_2_) is > 90%, and the respiratory rate is < 30 breaths/minute.^(36)^ If these criteria are not met or if the positive end-expiratory pressure > 10cmH_2_O, patient-ventilator asynchrony occurs, or rescue respiratory therapies are needed, an experienced medical team should be consulted before initiating rehabilitation.^(1)^ Moreover, the availability of adequate supplemental oxygen should be ensured, as unexpected demands may arise.^(32)^

Neurological status is a major factor in mobilization, especially in neurocritical care and stroke units. Low neurological tone (muscular flaccidity) or high neurological tone (spasticity) can complicate mobilization,^(37)^ but tools such as focal muscle vibration and non-immersive virtual reality may help.^(38-40)^ Although physical therapy generally does not affect cerebral hemodynamics,^(38)^ a low Glasgow coma scale score or poor consciousness can limit rehabilitation progress. Despite these challenges, mobilization remains crucial for patients with neurological injuries. Delirium, agitation, and pain also impact mobilization and should be carefully managed.^(31)^

Hematological and metabolic factors, along with subjective symptoms such as pain, shortness of breath, and emotional state, must also be considered. Monitoring physiological responses (e.g., heart rate, blood pressure, oxygenation) helps gauge tolerance to mobilization.^(37)^

In summary, ICU mobilization requires a comprehensive safety assessment based on objective parameters and multidisciplinary input to balance the risks and benefits.^(32)^ According to the European Respiratory Society (ERS) guidelines, mobilization should not be performed in patients who remain hemodynamically unstable despite vasopressor therapy or persistent desaturation despite supplemental oxygen.^(22)^ Adjusting pressure support or ventilator settings during mobilization may help prevent adverse events. Although in the TEAM trial, the mobilization strategy aimed to achieve the highest possible level of activity,^(11)^ current guidelines recommend progressive, stepwise mobilization programs to avoid overburdening patients.^(39)^

### Patient position

In critical care, patient positioning is a key component of mobility protocols. Proper positioning helps prevent complications such as pressure ulcers, muscle atrophy, and breathing disorders.^(41)^ Regularly changing patient positions and using supports such as pillows, cushions, and specialty mattresses can distribute pressure and maintain skin integrity.^(40)^ During rehabilitation, repositioning can also reduce discomfort and manage pain.^(11,42)^ However, a recent survey revealed that 47% of respondents found it challenging to reposition invasively-ventilated patients every two hours. Environmental and educational barriers affect positioning practices, with semirecumbent and side-lying positions being more common, whereas prone and head-down tilt positions are used less frequently.^(43)^

### Functional mobilization

Functional mobilization in the ICU is crucial, as some researchers argue that prolonged bed rest is anatomically, physiologically, and psychologically detrimental.^(44)^ Early functional mobilization, including sitting out of bed and walking beginning on the first postoperative day, has been shown to reduce the number of days in bed, shorten the ICU stay, and decrease functional deterioration. This shift in ICU culture has led to a focus on functional mobilization of critically ill patients.^(45)^

Intensive care unit mobilization includes a range of activities, including passive and active range-of-motion exercises, resistance training, positioning, functional mobility and transfers, respiratory muscle training, neuromuscular electrical stimulation, and the use of tilt tables and cycle ergometers.^(46)^ While interest in nonvoluntary exercises such as neuromuscular electrical stimulation and passive cycle ergometry is increasing, functional exercises remain essential for all wakeful patients.^(46)^ Passive exercise is particularly beneficial when patients are not yet cooperative.^(41)^

### Documentation of outcomes

**Figure 2:**
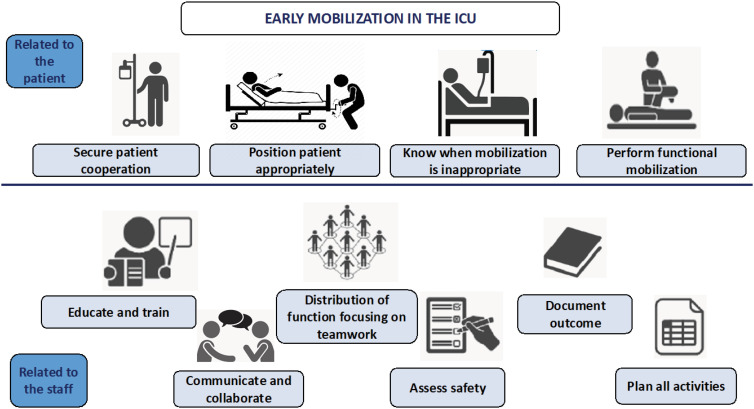
Ten recommendations for early mobilization in the intensive care unit.

Accurate documentation is crucial for ensuring a safe, efficient, and high-quality ICU mobilization protocol. First, it allows clinical teams to objectively assess individual responses to rehabilitation using scales that measure muscle strength, physical functioning, and respiratory capacity. This information is vital for tracking patient progress and adjusting the mobilization plan as needed.^(1)^ Second, documentation contributes to scientific evidence by evaluating effectiveness of the intervention, identifying factors associated with improved outcomes, and supporting evidence-based practice development. Finally, thorough documentation provides clear information on patient recovery progress, facilitating informed decision-making for patients, their families, and the ICU team. It also serves as an educational resource, helping patients and families understand rehabilitation expectations and potential long-term outcomes. The ICU Mobility Scale, which focuses on active mobilization, is a valuable tool for documenting active rehabilitation.^(46)^ Recommendations for safe mobilization are summarized in figure 2 and detailed in table 1.

This figure provides ten key recommendations for implementing early mobilization in the ICU to optimize patient outcomes and safety.

ICU - intensive care unit.

**Table 1:** Key strategies for encouraging early mobilization in critical care settings

	Comments
Education and training	Knowledge, attitudes, and perceptions related to early mobilization in the intensive care unit remain insufficient among professionals. Systematic implementation of education and training strategies on physical therapy for intensive care unit staff is essential.
Communication and collaboration	Effective communication and collaboration among intensive care unit staff are crucial and should be characterized by: Fluid and continuous communication Involvement of patients and families Patient-centered care Ongoing training and education
Initial action: patient screening	It should be recognized that not all patients are suitable candidates for mobilization. Continuous evaluation is necessary to determine the appropriate timing for starting mobilization. classical contraindications should be considered, including: Myocardial infarction Active bleeding Increased and unstable intracranial pressure Unstable pelvic fractures
Planning of activities	Appropriate planning should consider patient-related, staff-related, resource-related, and structural factors.
Distribution of functions focused on teamwork	Physicians involved in the decision to initiate mobilization should not be limited to intensivists; internists and fellows are increasingly involved in this process. Physical therapists lead the promotion of physical mobilization, while nurses manage invasive devices (e.g., central venous catheters or arterial lines) and address pain, *delirium*, and agitation. Speech and language therapists, along with occupational therapists, focus on cognitive rehabilitation and other goals.
Patient cooperation	For this evaluation, experts recommend using the "Standardized Five Questions" scale. This tool uses five straightforward questions to assess the subject's level of cooperation.
Safety assessment	Objective safety assessment should include evaluating hemodynamics, respiratory function and airways, and neurological status.
Patient position	In the critical care setting, changing patient position is a key component of rehabilitation and mobility protocols.
Functional mobilization	In the intensive care unit, mobilization techniques include passive range-of-motion exercises, active range-of-motion exercises, resistance training, positioning, functional mobility and transfers, respiratory muscle training, neuromuscular electrical stimulation, and tilt-table and cycle ergometer exercises.
Documentation of outcomes	Documenting the rehabilitation process is crucial for providing clear and accurate updates on the patient's recovery. This documentation supports informed decision-making by the patient, their family, and the intensive care unit team.

Source: Based on: Hodgson CL, Schaller SJ, Nydahl P, Timenetsky KT, Needham DM. Ten strategies to optimize early mobilization and rehabilitation in intensive care. Crit Care. 2021;25(1):324.

### Summary of evidence

Based on the reviewed evidence, mobilization in critically ill patients can mitigate the detrimental effects of an ICU stay.^(8)^ These benefits, however, depend on the implementation of tailored physical therapy protocols and the coordination of interdisciplinary teams that emphasize high levels of organization and effective communication.^(21)^

While there is some debate about the impact of mobilization on short- and long-term patient survival and mortality, it is important to recognize that ICU mortality is influenced by multiple factors, making it difficult to attribute outcomes solely to physical therapy. The TEAM study reported adverse events associated with maximal physical therapy, particularly in the early stages.^(11)^ However, physical therapy remains widely considered a safe and cost-effective approach for reducing complications related to hospitalization.^(11)^

## CONCLUSION

Early mobilization and rehabilitation protocols that incorporate individualized evaluation, clinical monitoring, physical exercise, patient and family engagement, and multidisciplinary teamwork are essential components of comprehensive intensive care unit management. By prioritizing functional mobility and involving all stakeholders in the rehabilitation process, healthcare providers can achieve better patient outcomes, improve the overall intensive care unit experience, and reduce both short- and long-term complications.

## References

[1] Hodgson CL, Stiller K, Needham DM, Tipping CJ, Harrold M, Baldwin CE (2014). Expert consensus and recommendations on safety criteria for active mobilization of mechanically ventilated critically ill adults. Crit Care.

[2] Needham DM, Davidson J, Cohen H, Hopkins RO, Weinert C, Wunsch H (2012). Improving long-term outcomes after discharge from intensive care unit. Crit Care Med.

[3] Taylor C (2024). Intensive care unit-acquired weakness. Anaesth Intensive Care Med.

[4] Gutenbrunner C, Kubat B, Kröhn S, Haller H, Schiller J, Korallus C (2021). Teaching functioning, disability and rehabilitation to first year medical students. J Rehabil Med.

[5] Bloch S, Polkey MI, Griffiths M, Kemp P (2012). Molecular mechanisms of intensive care unit-acquired weakness. Eur Respir J.

[6] Fazzini B, Battaglini D, Carenzo L, Pelosi P, Cecconi M, Puthucheary Z (2022). Physical and psychological impairment in survivors of acute respiratory distress syndrome: a systematic review and meta-analysis. Br J Anaesth.

[7] Daum N, Drewniok N, Bald A, Ulm B, Buyukli A, Grunow JJ (2024). Early mobilisation within 72 hours after admission of critically ill patients in the intensive care unit: a systematic review with network meta-analysis. Intensive Crit Care Nurs.

[8] Denehy L, Lanphere J, Needham DM (2017). Ten reasons why ICU patients should be mobilized early. Intensive Care Med.

[9] Lang JK, Paykel MS, Haines KJ, Hodgson CL (2020). Clinical practice guidelines for early mobilization in the ICU: a systematic review. Crit Care Med.

[10] Eggmann S, Timenetsky KT, Hodgson C (2024). Promoting optimal physical rehabilitation in ICU. Intensive Care Med.

[11] Hodgson CL, Bailey M, Bellomo R, Brickell K, Broadley T, Buhr H, TEAM Study Investigators and the ANZICS Clinical Trials Group (2022). Early active mobilization during mechanical ventilation in the ICU. N Engl J Med.

[12] Zhang H, Liu H, Li Z, Li Q, Chu X, Zhou X (2021). Early mobilization implementation for critical ill patients: a cross-sectional multi-center survey about knowledge, attitudes, and perceptions of critical care nurses. Int J Nurs Sci.

[13] Dagnachew TK, Woldegerima Berhe Y, Yalew Mustofa S, Birlie Chekol W (2023). Clinicians' knowledge and attitude towards early mobilization in intensive care units in Ethiopian tertiary hospitals: a multi-centre study. SAGE Open Med.

[14] de Souza PN, Kroth JB, dos Santos Ligero A, Mendes JM, Maida ALV, Pastore L (2022). Effectiveness of a quality improvement strategy with implementation of a specific visual tool to promote ICU early mobilization. Sci Rep.

[15] Zhang L, Hu W, Cai Z, Liu J, Wu J, Deng Y (2019). Early mobilization of critically ill patients in the intensive care unit: a systematic review and meta-analysis. PLoS One.

[16] De Rosa S, Battaglini D, Bennett V, Rodriguez-Ruiz E, Zaher AM, Galarza L, Schaller SJ, NEXT Committee of the ESICM (2023). Key steps and suggestions for a promising approach to a critical care mentoring program. J Anesth Analg Crit Care.

[17] Fitzgerald KG, Tipton E (2024). A knowledge mobilization framework: toward evidence-based statistical communication practices in education research. J Res Educ Eff.

[18] Haines KJ (2018). Engaging families in rehabilitation of people who are critically ill: an underutilized resource. Phys Ther.

[19] Mezidi M, Guérin C (2018). Effects of patient positioning on respiratory mechanics in mechanically ventilated ICU patients. Ann Transl Med.

[20] Au SS, Roze des Ordons AL, Amir Ali A, Soo A, Stelfox HT (2019). Communication with patients' families in the intensive care unit: a point prevalence study. J Crit Care.

[21] Hickmann CE, Castanares-Zapatero D, Bialais E, Dugernier J, Tordeur A, Colmant L (2016). Teamwork enables high level of early mobilization in critically ill patients. Ann Intensive Care.

[22] Adler J, Malone D (2012). Early mobilization in the intensive care unit: a systematic review. Cardiopulm Phys Ther J.

[23] Devlin JW, Skrobik Y, Gélinas C, Needham DM, Slooter AJ, Pandharipande PP (2018). Clinical Practice Guidelines for the Prevention and Management of Pain, Agitation/Sedation, Delirium, Immobility, and Sleep Disruption in Adult Patients in the ICU. Crit Care Med.

[24] Green M, Marzano V, Leditschke IA, Mitchell I, Bissett B (2016). Mobilization of intensive care patients: a multidisciplinary practical guide for clinicians. J Multidiscip Healthc.

[25] Sacomori C, Lorca LA, Martinez-Mardones M, Salas-Ocaranza RI, Reyes-Reyes GP, Pizarro-Hinojosa MN (2021). A randomized clinical trial to assess the effectiveness of pre- and post-surgical pelvic floor physiotherapy for bowel symptoms, pelvic floor function, and quality of life of patients with rectal cancer: CARRET protocol. Trials.

[26] Ervin JN, Kahn JM, Cohen TR, Weingart LR (2018). Teamwork in the intensive care unit. Am Psychol.

[27] Patterson ES, Roth EM, Woods DD, Chow R, Gomes JO (2004). Handoff strategies in settings with high consequences for failure: lessons for health care operations. Int J Qual Health Care.

[28] Semmons J (2022). The role of specialist physiotherapy in a pain management clinic – traditional and novel approaches. Anaesth Intensive Care Med.

[29] Miranda Rocha AR, Martinez BP, Maldaner da Silva VZ, Forgiarini LA (2017). Early mobilization: why, what for and how?. Med Intensiva.

[30] Battaglini D, Ciaravolo E, Caiffa S, Delpiano L, Ball L, Vena A, Giacobbe DR, Bassetti M, Matta B, Pelosi P, Robba C, GECOVID Collaborators (2023). Systemic and cerebral effects of physiotherapy in mechanically ventilated subjects. Respir Care.

[31] Stiller K, Phillips A (2003). Safety aspects of mobilising acutely ill inpatients. Physiother Theory Pract.

[32] Woodbridge HR, McCarthy CJ, Jones M, Willis M, Antcliffe DB, Alexander CM (2024). Assessing the safety of physical rehabilitation in critically ill patients: a Delphi study. Crit Care.

[33] Hanada M, Tawara Y, Miyazaki T, Sato S, Morimoto Y, Oikawa M (2017). Incidence of orthostatic hypotension and cardiovascular response to postoperative early mobilization in patients undergoing cardiothoracic and abdominal surgery. BMC Surg.

[34] Stiller K, Phillips A, Lambert P (2004). The safety of mobilisation and its effect on haemodynamic and respiratory status of intensive care patients. Physiother Theory Pract.

[35] Castelli L, Iacovelli C, Fusco A, Amoruso V, Cuccagna C, Loreti C (2023). The role of technological rehabilitation in patients with intensive care unit weakness: a randomized controlled pilot study. J Clin Med.

[36] Aprile I, Iacovelli C, Pecchioli C, Cruciani A, Castelli L, Germanotta M (2020). Efficacy of focal muscular vibration in the treatment of upper limb spasticity in subjects with stroke outcomes: randomized controlled trial. J Biol Regul Homeost Agents.

[37] Calabrò RS, Naro A, Russo M, Milardi D, Leo A, Filoni S (2017). Is two better than one? Muscle vibration plus robotic rehabilitation to improve upper limb spasticity and function: a pilot randomized controlled trial. PLoS One.

[38] Schaller SJ, Scheffenbichler FT, Bein T, Blobner M, Grunow JJ, Hamsen U (2024). Guideline on positioning and early mobilisation in the critically ill by an expert panel. Intensive Care Med.

[39] Ippolito M, Cortegiani A, Biancofiore G, Caiffa S, Corcione A, Giusti GD (2022). The prevention of pressure injuries in the positioning and mobilization of patients in the ICU: a good clinical practice document by the Italian Society of Anesthesia, Analgesia, Resuscitation and Intensive Care (SIAARTI). J Anesth Analg Crit Care.

[40] Thomas PJ, Paratz JD, Stanton WR, Deans R, Lipman J (2006). Positioning practices for ventilated intensive care patients: current practice, indications and contraindications. Aust Crit Care.

[41] Zuo XL, Meng FJ (2015). A care bundle for pressure ulcer treatment in intensive care units. Int J Nurs Sci.

[42] Powers JH (1944). The abuse of rest as a therapeutic measure in surgery. Early postoperative activity and rehabilitation. JAMA.

[43] Yang X, Zhang T, Cao L, Ye L, Song W (2023). Early mobilization for critically ill patients. Respir Care.

[44] Dubb R, Nydahl P, Hermes C, Schwabbauer N, Toonstra A, Parker AM (2016). Barriers and strategies for early mobilization of patients in intensive care units. Ann Am Thorac Soc.

[45] Chapple LA, Parry SM, Schaller SJ (2022). Attenuating muscle mass loss in critical illness: the role of nutrition and exercise. Curr Osteoporos Rep.

[46] Hodgson CL, Bailey M, Bellomo R, Berney S, Buhr H, Denehy L, Gabbe B, Harrold M, Higgins A, Iwashyna TJ, Papworth R, Parke R, Patman S, Presneill J, Saxena M, Skinner E, Tipping C, Young P, Webb S, Trial of Early Activity and Mobilization Study Investigators (2016). A binational multicenter pilot feasibility randomized controlled trial of early goal-directed mobilization in the ICU. Crit Care Med.

